# Co-alterations of circadian clock gene transcripts in human placenta in preeclampsia

**DOI:** 10.1038/s41598-022-22507-3

**Published:** 2022-10-25

**Authors:** Guoli Zhou, Emily Winn, Duong Nguyen, Eric P. Kasten, Margaret G. Petroff, Hanne M. Hoffmann

**Affiliations:** 1 Clinical & Translational Sciences Institute, Michigan State University, 909 Wilson Rd. Suite B500, East Lansing, MI 48824 USA; 2 Department of Pathobiology and Diagnostic Investigation, Michigan State University, East Lansing, MI 48824 USA; 3 Department of Animal Science, Reproductive and Developmental Science Program and Neuroscience Program, College of Agriculture and Natural Resources, Michigan State University, Interdisciplinary Science and Technology Building #3010, 766 Service Road, East Lansing, MI 48824 USA; 4 Department of Radiology, Michigan State University, East Lansing, MI 48824 USA; 5 Department of Microbiology and Molecular Genetics, College of Veterinary Medicine, Michigan State University, East Lansing, MI 48824 USA

**Keywords:** Computational biology and bioinformatics, Data mining, Gene regulatory networks, Microarrays, Cell migration

## Abstract

Pre-eclampsia (PE) is a hypertensive condition that occurs during pregnancy and complicates up to 4% of pregnancies. PE exhibits several circadian-related characteristics, and the placenta possesses a functioning molecular clock. We examined the associations of 17 core circadian gene transcripts in placenta with PE vs. non-PE (a mixture of pregnant women with term, preterm, small-for-gestational-age, or chorioamnionitis) using two independent gene expression datasets: GSE75010-157 (80 PE vs. 77 non-PE) and GSE75010-173 (77 PE and 96 non-PE). We found a robust difference in circadian gene expression between PE and non-PE across the two datasets, where *CRY1* mRNA increases and *NR1D2* and *PER3* transcripts decrease in PE placenta. Gene set variation analysis revealed an interplay between co-alterations of circadian clock genes and PE with altered hypoxia, cell migration/invasion, autophagy, and membrane trafficking pathways. Using human placental trophoblast HTR-8 cells, we show that CRY1/2 and NR1D1/2 regulate trophoblast migration. A subgroup study including only term samples demonstrated that *CLOCK*, *NR1D2*, and *PER3* transcripts were simultaneously decreased in PE placenta, a finding supported by CLOCK protein downregulation in an independent cohort of human term PE placenta samples. These findings provide novel insights into the roles of the molecular clock in the pathogenesis of PE.

## Introduction

Pre-eclampsia (PE) is a hypertensive condition of pregnancy and complicates up to 4% of pregnancies in the US^1,2^. PE is a leading cause of maternal, fetal, and neonatal morbidity and mortality^3,4^. Women with a history of PE have an increased risk of cardiovascular diseases, type II diabetes, and chronic kidney disease later in life^3,5^. The involved risk factors for pregnant women to develop PE include: a history of preeclampsia, chronic hypertension, pregestational diabetes mellitus, multiple pregnancy, antiphospholipid syndrome, obesity, advanced maternal age, nulliparity, history of chronic kidney disease, use of assisted reproductive technologies, and genetic susceptibility^3,6,7^. PE is typically defined as the presence of hypertension and either proteinuria or signs of severe multiorgan dysfunction after 20 weeks' gestation in a previously normotensive woman^3,8^. Based on the timing of disease onset, PE can be grouped into two subtypes: early-onset (occurring at < 34 weeks' gestation) and late-onset (occurring at ≥ 34 weeks' gestation)^9^. The two subtypes of PE may have different clinical characteristics and pathophysiology^10^, as well as different associated risk factors and outcomes^9,11^. However, the stratification of PE by the timing of disease onset still cannot fully explain the heterogeneity of PE’s clinical presentation, which might at least partially account for the lack of robust predictive biomarkers and effective treatments. To date, the only effective treatment for PE still remains the delivery of the placenta and fetus^3,4^, although low-dose aspirin has been recommended for reducing the risk of PE in high-risk pregnant women^8,12^ and novel potential therapies are emerging^8,13,14^.

Light-induced circadian dysregulation has been associated with multiple pregnancy complications including miscarriage^15–17^, preterm birth^17,18^, and gestational diabetes^19^. There is compelling evidence that pathophysiological functions regulated by the circadian system contribute to PE, where pregnant women with PE frequently are diagnosed with an abnormal circadian blood pressure rhythm^20^. The abnormal blood pressure associated with PE is often referred to as ‘non-dipping nocturnal high blood pressure’, in which blood pressure drops by less than 10% at night^21^. Pregnant women with early-onset PE present with a significantly higher prevalence of non-dipping nocturnal hypertension compared to late-onset PE^22^. Meanwhile, in women who had PE with severe features, non-dipping nocturnal blood pressure was associated with adverse maternal/perinatal outcomes such as preterm birth, retinopathy, HELLP (Hemolysis, Elevated Liver enzymes and Low Platelets) syndrome, low birth weight, and fetal growth restriction^23^. In addition, in a randomized chronotherapy trial, where the time-of-day of drug intake was recorded and controlled for, administration of low-dose aspirin at bedtime significantly reduced the ambulatory blood pressure and the incidence of preeclampsia, as compared to the ingestion of aspirin in the morning^24^. Together, these data suggest that at least some subtypes and/or specific clinical presentations of PE are related to circadian rhythms. Whether these circadian-related aspects of PE are controlled by either maternal central or placental peripheral clock systems or both remain unknown.

Preeclampsia is caused by placental dysfunction^3,8,25^. Studies on global placental gene expression profiling have significantly improved our understanding of the heterogeneity of PE’s clinical presentations, etiology, and pathophysiology^26–30^. However, our current understanding of placental PE-related gene expression lacks studies on the potential role of clock genes. This is surprising, since PE has numerous circadian-related characteristics and the placenta possesses a functioning molecular clock, which drives endogenous cellular circadian rhythms^31–36^. At the cellular level, circadian rhythms are generated by a complex cell-autonomous transcription-translation feedback mechanism, generating 24 h gene expression rhythms. Among the core circadian transcriptional regulators are: Aryl hydrocarbon receptor nuclear translocator-like protein 1 (ARNTL1, also known as BMAL1), Circadian Locomotor Output Cycles Kaput (CLOCK), Period 1/2/3 (PER1/2/3), Cryptochrome 1/2 (CRY1/2) and the nuclear receptor subfamily 1 group D members 1/2 (NR1D1/2, also known as REV-ERBα/β). Circadian clock genes are expressed in female reproductive tissues, such as the ovary, oviducts, uterus, and placenta^33,37–42^. While the role of circadian genes in the ovary is well-understood^43^, their role in the placenta remains elusive.

The goal of this study is to identify specific clock genes associated with PE by examining the mRNA expression patterns of 17 core circadian transcripts in PE and non-PE placentas. Using existing gene expression datasets together with functional and molecular approaches, we identified differentially expressed circadian genes in PE placentas whose expression affects trophoblast migration in vitro. These results will allow future studies to address the role of these clock genes in placental development and function for the etiology/pathogenesis and therapeutics of PE.

## Materials and methods

### Human research

All methods were performed in accordance with the relevant guidelines and regulations. According to the IRB guidelines and the HIPPA Privacy Rule, the analysis of de-identified, publicly available data does not constitute human subjects research as defined at 45 CFR 46.102 and thus, the present study does not require IRB review. Placentas for molecular analysis were collected in accordance with protocols approved by the Institutional Review Boards of Michigan State University, Mount Sinai Hospital, and the University of Kansas Medical Center. Placentas from gestational age- and delivery-matched healthy and PE pregnancies were collected from the Research Center for Women’s and Infant’s Health BioBank at the Samuel Lunenfeld Research Institute (Mount Sinai Hospital, Toronto, Ontario, Canada).

### Study design, selection of patients, and demographics

Publicly available human placenta microarray gene expression data (GSE75010, released in 2016) from the National Center for Biotechnology Information (NCBI) Gene Expression Omnibus (GEO) were analyzed. The GSE75010 data were composed of two independent datasets. One dataset, GSE75010-157, was a PE microarray dataset generated from a total of 157 placenta samples (80 PE vs. 77 non-PE) that were obtained from the Research Centre for Women’s and Infants’ Health BioBank (Mount Sinai Hospital, Toronto, Canada)^44^. The second dataset, GSE75010-173, was a combination of 173 placental samples (77 PE vs. 96 non-PE) from seven independent studies conducted in five different countries (Canada, China, Japan, Norway, US)^44^.

For the dataset ‘GSE75010-157’, PE was defined as the onset of systolic pressure ≥ 140 mmHg and/or diastolic pressure ≥ 90 mmHg after the 20th week of gestation, with proteinuria (> 0.3 g protein/day, or ≥ 2 + on dipstick)^44^. Fetal sex, preterm (< 34 weeks gestation), and small-for-gestational-age infants (SGA, neonatal birth weight < 10th percentile for gestational age and sex) were approximately balanced between PE and non-PE groups. Two groups (PE vs. non-PE) were designated as ‘Overall PE’ vs. ‘Overall non-PE’ due to such heterogeneity of the patients’ composition. Only samples from singleton pregnancies were included, and patients with pre-existing or gestational diabetes, sickle cell anemia, or severe obesity (BMI ≥ 40) were excluded. The PE group was composed of 44.9% of Caucasian, 75.0% with maternal age ≥ 30 years old, 18.2% having obesity before pregnancy (30 < BMI < 40), and 21.3% having previous miscarriage (Table S1). There were no significant differences in these maternal characteristics between PE and non-PE groups (Table S1).

For the dataset ‘GSE75010-173’, among seven different studies^45–51^, four of them used the definition of PE similar to that in the dataset ‘GSE75010-157’; two studies used severe PE only, which was defined as blood pressure of at least 160 mmHg (systolic) and/or 110 mmHg (diastolic) after the 20th week of gestation, with proteinuria (> 2 g protein/day, or > 2 + on dipstick); one study used both^44^. Patients in the PE group consisted of 10.4% from Canada, 14.3% from China, 23.4% from Japan, 22.1% from Norway, and 29.9% from the US (Table S2).

### Placental tissue microarray gene expression data

The dataset ‘GSE75010-157’ was generated with the Human Gene 1.0 ST Array chips (Affymetrix). The dataset ‘GSE75010-173’ was a combination of the microarray data from seven different studies, in which five different commercial microarray kits from Applied Biosystems, Agilent Technologies, Affymetrix, Roche NimbleGen Arrays, and Illumina were applied^44^. According to Leavey et al., both datasets were harmonized by normalization and batch effect correction with the virtual Array R package^52^ to improve the reproducibility of the downstream statistical analyses. Finally, all data were converted into log2 values using R Affy library^53^. A total of 14,651 genes with expression values were detectable in both datasets^44^. The harmonized datasets ‘GSE75010-157’ and ‘GSE75010-173’ were used as discovery set and replication set, respectively.

### Bioinformatics and statistical analyses

**Figure 1:**
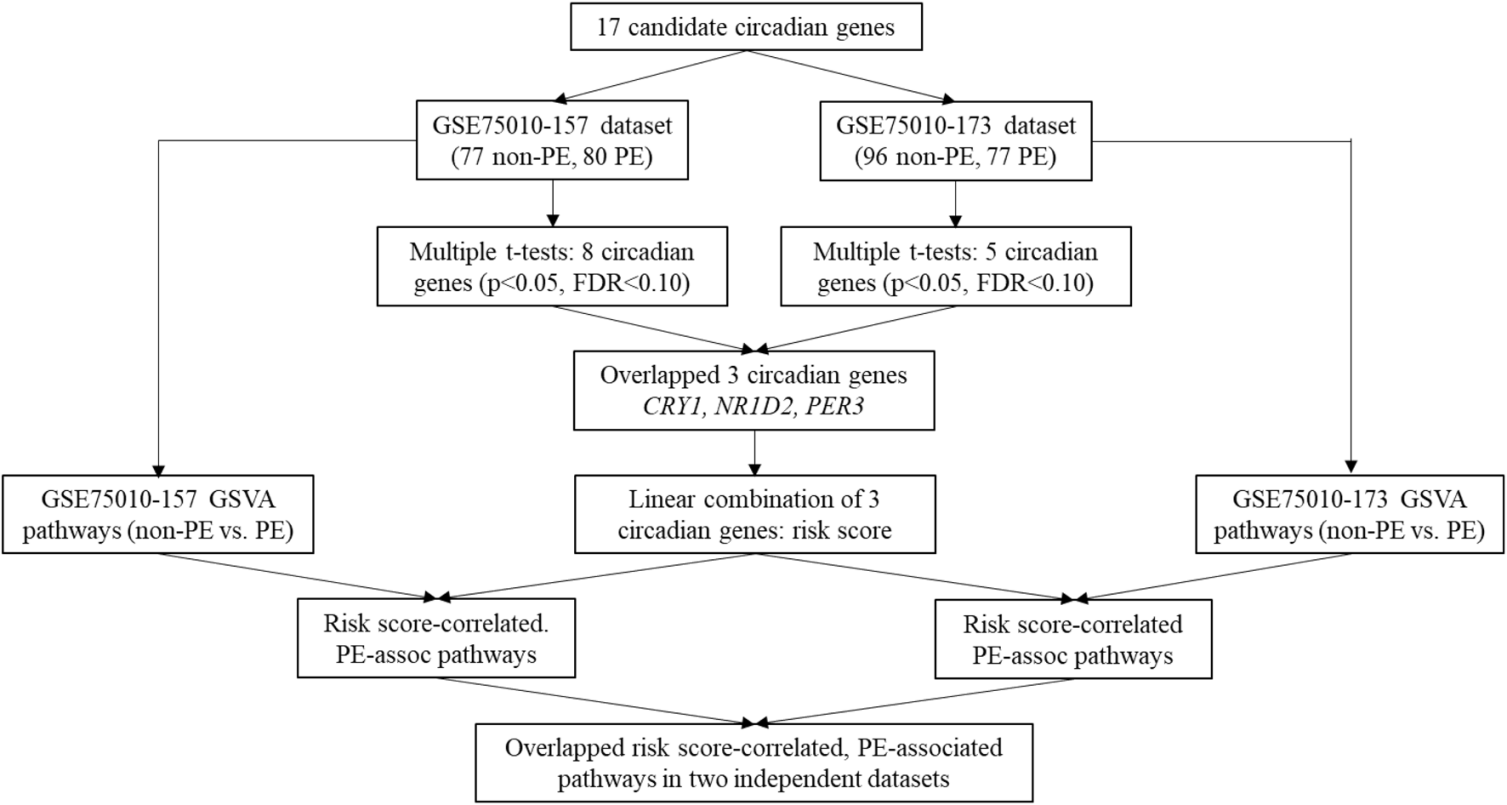
Bioinformatics pipeline to analyze circadian genes-correlated, PE-associated pathways.

A bioinformatics pipeline that we developed to identify PE-associated circadian genes and their correlated pathways with two independent datasets is presented in Fig. 1. Briefly, we retrieved 17 core circadian transcripts (*ARNTL*, *ARNTL2*, *CLOCK*, *CRY1*, *CRY2*, *NPAS1*, *NPAS2*, *NPAS3*, *NR1D1*, *NR1D2*, *PER1*, *PER2*, *PER3*, *RORA*, *RORB*, *RORC*, and *TIMELESS*) from two independent datasets (GSE75010-157 and GSE75010-173). We selected these core clock genes based on their critical roles in circadian rhythms^54^ and the detectability of their mRNAs in the available microarray data. We summarized the mRNA levels (log2-transformed) of all 17 core circadian genes in placenta as means with standard deviations (SDs). The mean differences in gene mRNA levels between the non-PE and PE groups were examined with a two-sample, two-tailed t-test and a false discovery rate (FDR) to adjust for multiple comparisons. The cut-off values for statistical significance were set as p < 0.05 and FDR q < 0.10^55^. Then, we overlapped the two pools of significant candidate genes identified from two independent datasets and obtained common significant PE-associated circadian genes across two datasets (Fig. 1). By referring to our previous method^56^, we linearly combined these common significant PE-associated circadian genes to generate a risk score of PE (Fig. 1). A Firth binary logistic regression model^57^ was applied to assess the association strength between this risk score (categorized into a binary variable using the median of the risk score as a cut-off value) and PE.

Next, with our previous methods^56^, we conducted the Gene Set Variation Analysis (GSVA) using two independent datasets to explore the biological pathways that are involved in PE-clock gene relationship, respectively (Fig. 1). The pathway sub-collection—c2.cp.v7.4.symbols.gmt in the Molecular Signatures Database (MSigDB) was used. We applied limma R package^58^ to identify differential pathways between non-PE and PE with the cut-offs of p < 0.05 and Benjamini and Hochberg adjusted p < 0.10 (Fig. 1). Then, we further examined the correlations of the identified clock genes-based risk score with the PE-associated pathways with Pearson Correlation statistic (p < 0.05 and Benjamini and Hochberg adjusted p < 0.10) (Fig. 1). Finally, two pools of PE-associated pathways correlated with circadian genes-based risk score were overlapped to generate common PE-associated pathways that were correlated with the circadian genes-based risk score (Fig. 1).

PE can be divided into preterm PE and term PE^59,60^. To disentangle the possible confounding effect of preterm birth (PTB, defined as birth before 37 weeks of gestation) on the relationship between circadian clock genes and PE, a subgroup analysis was performed by removing the individuals who were diagnosed as PTB from overall PE and non-PE groups in the dataset GSE75010-157. There was no available information about PTB diagnosis in the dataset GSE75010-173. The analytic methods for identifying PE-associated circadian genes and their correlated pathways after removing patients diagnosed with PTB were the same as those used in the analyses of the GSE75010-157 dataset.

We conducted all data management and statistical analyses described above using R (R Development Core Team) and SAS v9.4 (SAS Institute, Cary, North Carolina).

### HTR-8 cell culture and wound healing assay

Trophoblast cells derived from a 6–12 weeks-old human placenta (HTR-8 SVNeo, referred to as HTR-8 cells, ATCC #CRL-3271) were cultured in RPMI 1640 Medium (Gibco, #11875093) with 10% FBS (Sigma-Aldrich, #F4135) and 1% Penicillin–Streptomycin (Sigma-Aldrich Cat# P4333). HTR-8 cells were plated at 0.5 million cells/ml per well in a 24-well wound healing assay (Abcam, #ab242285). Twenty-four h after plating, the HTR-8 cells formed a monolayer and the inserts were removed, where after the cells were treated with vehicle (DMSO 1/500 or 1/5000 dilution, no difference in HTR-8 cell migration in response to DMSO concentration was found and data were pooled), KL001 (Tocris Bioscience, # 46-851-0), PF670462 (Tocris Bioscience™ Cat# 33-161-0), SR9009 (Tocris Bioscience, # 5855), SR8278 (Tocris Bioscience, # 4463), or KL101 (Sigma Aldrich, #SML3014) at 1 μM and 10 μM. Bright field image acquisition of the wound healing area was done using a light microscope (Leica DMi1) 0, 24 and 48 h after treatment. Data were analyzed using ImageJ/Fiji^®^ version 1.53 (NIH).

### Westernblot

Human placenta samples were matched for gestation age and mode of delivery (C-section, C-section + labor, Vaginal delivery + labor) between PE and normal placentas. After collection, samples were rapidly frozen in liquid nitrogen before being stored at − 80 °C until use. Proteins were extracted by homogenization for 15–30 s in RIPA buffer containing protease and phosphatase inhibitors using a Bead Beater (MP Biomedicals, Irvine, CA, USA). Lysates were centrifuged for 10–20 min at 4 °C, 15,000*g*. Supernatant was recovered and protein concentration determined using the Pierce BCA Assay (Thermo Scientific, Waltham, MA, USA). Proteins were then denatured by boiling in Laemmli buffer at 95 °C for 5 min, and 20 µg were electrophoresed on a 10% SDS–polyacrylamide gel at 120 V for 45 min. Protein was then transferred to a nitrocellulose membrane (Cytiva Amersham™ Protran™; Fisher Scientific, Pittsburgh, PA, USA) by electroblotting at 200 V for 45 min. The membrane was blocked in 3% milk for 1 h at RT and incubated with rabbit anti-human CLOCK primary antibody (1:1000, ab3517, Abcam, Cambridge, UK, RRID: AB_303886) for 16 h at 4 °C. After washing, the blot was incubated in goat anti-rabbit IgG-HRP secondary antibody (1:2000, BioRad, 1706515, RRID:AB_11125142) in 3% milk for 1 h at RT. HRP activity of bound secondary antibody was visualized in an iBright (ThermoFisher Scientific) using Enhanced Chemiluminescent reagent (Amersham) according to the manufacturer’s instructions. To stain for beta ACTIN, the membrane was stripped with stripping buffer (Restore Western Blot Stripping Buffer, 21059, Thermo Scientific), washed 3 × 5 min in TBS and 1 × 5 min in TBS-Tween 0.05%, blocked with buffer containing 3% milk for 1 h at RT, and incubated with a rabbit anti-beta ACTIN antibody (1:2000 Invitrogen, MA5-32479, RRID: AB_2809756). Secondary antibody and visualization with ECL were similar to those for CLOCK antibody. We verified the proteins by their molecular weight using 10 µl of a protein marker (Precision Plus Protein Standards, Bio-Rad, 161-0374). We confirmed the used CLOCK antibody had non-specific bands below 50 kDa. These non-specific bands did not interfere with CLOCK analysis at 100 kDa. Using Image J (NIH), the integral densities of CLOCK protein bands were normalized to the integral density of beta-ACTIN.

## Results

### The clock genes CRY1, NR1D2, and PER3 are differentially expressed in the placentas of preeclamptic patients in two independent datasets

To determine if expression of the 17 candidate circadian genes is dysregulated in PE placentas, we analyzed their expression using two independent publicly available datasets, comparing placentas from PE versus non-PE patients. To find the differentially expressed circadian genes that commonly exist in two datasets, we overlapped the results from the separate analyses with two datasets. We found that the expression of *CRY1*, *NR1D2*, and *PER3* gene transcripts is robustly different between overall PE and non-PE across the two datasets (Table 1). The average expression of *NR1D2* and *PER3* transcripts in placenta is lower in PE than in non-PE across the two datasets, respectively (the decrease percentages are 2.4%-4.0% for *NR1D2* and 3.0%-3.6% for *PER3* genes in the two datasets, all p ≤ 0.0030 and FDR ≤ 0.0225, Table 1). In contrast, the mean of *CRY1* gene transcript in placenta is significantly higher in PE than in non-PE (the increase percentages are 1.5–1.7%, p ≤ 0.0250 and FDR ≤ 0.0711, Table 1). The remaining circadian gene transcripts are not significantly different between PE vs. non-PE in either dataset (e.g., *ARNTL*, *CRY2*, *NPAS2*, *NPAS3*, *NR1D1*, *PER1*, and *RORC*, p > 0.05 and FDR > 0.10), or are different in one, but not the other dataset (e.g., *ARNTL2*, *NPAS1*, *PER2*, *RORB*, and *TIMELESS* are different in GSE75010-157, but not in GSE75010-173; *CLOCK* and *RORA* are the opposite, Table 1).

**Table 1:** Descriptive statistics of 17 candidate circadian clock genes’ expression levels in placenta from PE vs. non-PE, independent of gestation length (overall PE vs overall non-PE, respectively), in two independent datasets.

Gene	GSE75010-157 samples						GSE75010-173 samples					
	Non-PE		PE		p^b^	FDR^c^	Non-PE		PE		p	FDR
	n	Mean^a^ (SD)	n	Mean (SD)			n	Mean^a^ (SD)	n	Mean (SD)		
*ARNTL*	77	7.36 (0.38)	80	7.38 (0.42)	0.7006	0.8507	96	7.38 (0.39)	77	7.35 (0.32)	0.5509	0.7128
*ARNTL2*	77	7.95 (0.40)	80	8.09 (0.42)	0.0346	0.0735	96	8.01 (0.41)	77	8.01 (0.43)	0.9995	0.9995
*CLOCK*	77	8.88 (0.50)	80	8.73 (0.53)	0.0766	0.1302	96	8.88 (0.53)	77	8.71 (0.40)	0.0181	0.0711
***CRY1***	**77**	**8.39 (0.41)**	**80**	**8.53 (0.39)**	**0.0250**	**0.0607**	**96**	**8.40 (0.40)**	**77**	**8.53 (0.36)**	**0.0209**	**0.0711**
*CRY2*	77	8.39 (0.27)	80	8.38 (0.28)	0.9098	0.9098	96	8.40 (0.29)	77	8.38 (0.26)	0.6289	0.7128
*NPAS1*	77	6.56 (0.36)	80	6.76 (0.39)	0.0008	0.0045	96	6.68 (0.44)	77	6.61 (0.45)	0.2943	0.5440
*NPAS2*	77	9.06 (0.37)	80	8.96 (0.32)	0.0759	0.1302	96	8.99 (0.38)	77	9.03 (0.32)	0.4382	0.6607
*NPAS3*	77	6.55 (0.42)	80	6.45 (0.37)	0.1226	0.1788	96	6.51 (0.40)	77	6.48 (0.39)	0.6276	0.7128
*NR1D1*	77	7.23 (0.40)	80	7.32 (0.34)	0.1262	0.1778	96	7.28 (0.36)	77	7.22 (0.41)	0.2638	0.5440
***NR1D2***	**77**	**8.80 (0.48)**	**80**	**8.45 (0.51)**	**< 0.0001**	**0.0008**	**96**	**8.70 (0.51)**	**77**	**8.49 (0.41)**	**0.0030**	**0.0255**
*PER1*	77	7.40 (0.37)	80	7.41 (0.47)	0.8812	0.9098	96	7.37 (0.44)	77	7.43 (0.39)	0.3200	0.5440
*PER2*	77	7.93 (0.38)	80	8.08 (0.32)	0.0096	0.0272	96	8.05 (0.33)	77	7.95 (0.36)	0.0737	0.2088
***PER3***	**77**	**7.30 (0.32)**	**80**	**7.04 (0.30)**	**< 0.0001**	**0.0008**	**96**	**7.25 (0.30)**	**77**	**7.03 (0.27)**	**< 0.0001**	**0.0017**
*RORA*	77	8.54 (0.43)	80	8.52 (0.40)	0.7598	0.8611	96	8.59 (0.42)	77	8.45 (0.36)	0.0204	0.0711
*RORB*	77	6.64 (0.67)	80	7.00 (0.93)	0.0063	0.0214	96	6.79 (0.67)	77	6.87 (0.82)	0.4664	0.6607
*RORC*	77	6.56 (0.35)	80	6.64 (0.35)	0.1711	0.2237	96	6.55 (0.39)	77	6.62 (0.38)	0.2341	0.5440
*TIMELESS*	77	7.92 (0.40)	80	7.73 (0.32)	0.0015	0.0064	96	7.82 (0.37)	77	7.83 (0.36)	0.9138	0.9709

^a^Mean of gene expression values, normalized and log2-transformed. The mean change percentages of 3 circadian genes’ expression levels are: for CRY1 gene, (8.53–8.39)*100/8.39 = 1.7% in GSE75010-157 Samples and (8.53–8.40)*100/8.40 = 1.5% in GSE75010-173 Samples; for NR1D2 gene, (8.80–8.45)*100/8.80 = 4.0% in GSE75010-157 Samples and (8.70–8.49)*100/8.70 = 2.4% in GSE75010-173 Samples; and for PER3 gene, (7.30–7.04)*100/7.30 = 3.6% in GSE75010-157 Samples and (7.25–7.03)*100/7.25 = 3.0% in GSE75010-173 Samples.

^b^Two-sample, two-tailed t-tests were used to generate p-values.

Bold values denote statistical significance (p < 0.05 and FDR < 0.10) in both datasets.

### Association strength of the differentially expressed circadian genes-based risk score with overall PE in two independent datasets

To determine if co-alteration of the differentially expressed genes *CRY1, NR1D2,* and *PER3* occurs in PE placentas, we first generated a risk score of PE by using a linear combination of these 3 gene transcripts: Risk score = *CRY1* + (11-*NR1D2*) + (9-*PER3*), where the maximum values of NR1D2 and PER3 were < 11 and < 9, respectively. Then, we categorized the risk score into a binary variable using the median of the risk score as a cut-off value and calculated the odds-ratio (OR) of PE with the categorized risk score using a Firth binary logistic regression model. Maternal characteristic variables were not included in the model because they are similar between non-PE vs PE in GSE75010-157, and not available for GSE75010-173. As shown in Table 2, the OR (95% confidence interval) of PE for the risk score > median (< median as the reference) is 5.35 (2.70, 10.57) in GSE75010-157 and 5.46 (2.83, 10.54) in GSE75010-173 (both p values < 0.0001), respectively (Table 2). Collectively, these results show that co-alteration of *CRY1*, *NR1D2*, and *PER3* in the placenta is strongly associated with the risk of PE.

**Table 2:** Combined effect of the DE clock genes *CRY1, NR1D2* and *PER3* in placenta on the risk of overall PE with firth logistic regression model for two independent datasets.

Risk score*	N (%)	Non-PE, n (%)	PE, n (%)	OR_PE vs. Non-PE_ (95% CI)	p
**GSE75010-157**					
< Median	78 (100.0)	54 (69.2)	24 (30.8)	Ref	
> Median	79 (100.0)	23 (29.1)	56 (70.9)	**5.35 (2.70, 10.57)**	**< 0.0001**
**GSE75010-173**					
< Median	86 (100.0)	65 (75.6)	21 (24.4)	Ref	
> Median	87 (100.0)	31 (35.6)	56 (64.4)	**5.46 (2.83, 10.54)**	**< 0.0001**

*Risk score = *CRY1* + (11-*NR1D2*) + (9-*PER3*).

Bold values denote statistical significance (p < 0.05).

### PE-associated biological pathways correlate with CRY1, NR1D2, and PER3 in two independent datasets

We next used GSVA and Pearson correlation analyses to identify PE-associated pathways that correlate with co-alteration of *CRY1*, *NR1D2,* and *PER3*. We found 299 common PE-associated pathways that are correlated with the three circadian clock genes-based risk score, among which 212 are down-regulated and 87 are up-regulated across the two independent datasets (the range of absolute correlation coefficients is 0.15–0.57, all p < 0.05 and FDR < 0.10, Table S3).

To better interpret the most represented pathways that are up- and down-regulated in PE, we referred to the literature (regardless of cell origin and mammalian species). We manually grouped the top-most representative pathways into hypoxia-related and cell migration/invasion-related pathways (Table 3), where the top hypoxia-related pathways^7,61,62^ include galactose metabolism, hypoxia inducible factor (HIF) 1 transcription factor (TF) pathway, HIF2 pathway and Cori Cycle. The top identified cell migration/invasion-related pathways^8,63–68^ include transcriptional regulation by Runt-related transcription factor 1 (Runx1), deubiquitinating enzymes (DUBs), negative regulation of mesenchymal-epithelial transition factor (Met) activity, signaling by Notch4, β-catenin independent WNT signaling and PCP/CE polar (planar cell polarity/convergent extension) pathway, and signaling by Hedgehog and Hedgehog on State.

**Table 3:** Top 10 pathways correlated with *CRY1, NR1D2* and *PER3*-based risk score in placenta (overall PE vs. overall non-PE) in two independent datasets (cut-off: p < 0.05 and FDR < 0.10).

Risk score*-correlated pathway	GSE75010-157			GSE75010-173		
	r (95% CI)	p	FDR	r (95% CI)	p	FDR
**Top 10 decreased pathways in overall PE:**						
REACTOME_TRANSCRIPTIONAL_REGULATION_BY_RUNX1	−0.57 (−0.67, −0.45)	< 0.0001	0.0002	−0.36 (−0.49, −0.23)	< 0.0001	0.0009
REACTOME_SIGNALING_BY_HEDGEHOG	−0.54 (−0.64, −0.42)	< 0.0001	0.0002	−0.42 (−0.53, −0.29)	< 0.0001	0.0009
REACTOME_NEGATIVE_REGULATION_OF_MET_ACTIVITY	−0.53 (−0.63, −0.40)	< 0.0001	0.0002	−0.29 (−0.42, −0.14)	0.0001	0.0009
REACTOME_BETA_CATENIN_INDEPENDENT_WNT_SIGNALING	−0.51 (−0.62, −0.39)	< 0.0001	0.0002	−0.32 (−0.45, −0.18)	< 0.0001	0.0009
REACTOME_HEDGEHOG_ON_STATE	−0.50 (−0.61, −0.37)	< 0.0001	0.0002	−0.38 (−0.50, −0.24)	< 0.0001	0.0009
KEGG_BETA_ALANINE_METABOLISM	−0.50 (−0.61, −0.37)	< 0.0001	0.0002	−0.33 (−0.46, −0.19)	< 0.0001	0.0009
REACTOME_ANTIGEN_PROCESSING_CROSS_PRESENTATION	−0.50 (−0.60, −0.37)	< 0.0001	0.0002	−0.27 (−0.40, −0.13)	0.0003	0.0019
REACTOME_PCP_CE_PATHWAY	−0.49 (−0.60, −0.37)	< 0.0001	0.0002	−0.29 (−0.42, −0.15)	0.0001	0.0009
REACTOME_SIGNALING_BY_NOTCH4	−0.49 (−0.60, −0.36)	< 0.0001	0.0002	−0.30 (−0.43, −0.15)	< 0.0001	0.0009
REACTOME_METALLOPROTEASE_DUBS	−0.49 (−0.60, −0.36)	< 0.0001	0.0002	−0.25 (−0.38, −0.10)	0.001	0.0047
**Top 10 increased pathways in overall PE:**						
KEGG_GALACTOSE_METABOLISM	0.54 (0.42, 0.65)	< 0.0001	0.0002	0.39 (0.26, 0.51)	< 0.0001	0.0009
PID_HIF1_TFPATHWAY	0.52 (0.39, 0.62)	< 0.0001	0.0002	0.36 (0.22, 0.48)	< 0.0001	0.0009
WP_TRANSCRIPTIONAL_CASCADE_REGULATING_ADIPOGENESIS	0.50 (0.37, 0.61)	< 0.0001	0.0002	0.32 (0.18, 0.45)	< 0.0001	0.0009
KEGG_RIG_I_LIKE_RECEPTOR_SIGNALING_PATHWAY	0.49 (0.36, 0.60)	< 0.0001	0.0002	0.35 (0.22, 0.48)	< 0.0001	0.0009
REACTOME_TRANSCRIPTIONAL_REGULATION_OF_WHITE_ADIPOCYTE_DIFFERENTIATION	0.48 (0.35, 0.59)	< 0.0001	0.0002	0.23 (0.09, 0.37)	0.0019	0.0078
PID_P38_MKK3_6PATHWAY	0.46 (0.32, 0.57)	< 0.0001	0.0002	0.24 (0.10, 0.38)	0.0012	0.0054
WP_CORI_CYCLE	0.46 (0.33, 0.57)	< 0.0001	0.0002	0.38 (0.25, 0.50)	< 0.0001	0.0009
PID_HIF2PATHWAY	0.45 (0.32, 0.57)	< 0.0001	0.0002	0.30 (0.16, 0.43)	< 0.0001	0.0009
KEGG_ADIPOCYTOKINE_SIGNALING_PATHWAY	0.44 (0.31, 0.56)	< 0.0001	0.0002	0.30 (0.16, 0.43)	< 0.0001	0.0009
REACTOME_ACTIVATION_OF_AMPK_DOWNSTREAM_OF_NMDARS	0.44 (0.31, 0.56)	< 0.0001	0.0002	0.17 (0.02, 0.31)	0.0234	0.0515

*Risk score = *CRY1* + (11-*NR1D2*) + (9-*PER3*).

### Pharmacological stabilization of CRY1/2 and antagonism of NR1D1/2 slows down migration of the human trophoblast cell line HTR-8

**Figure 2:**
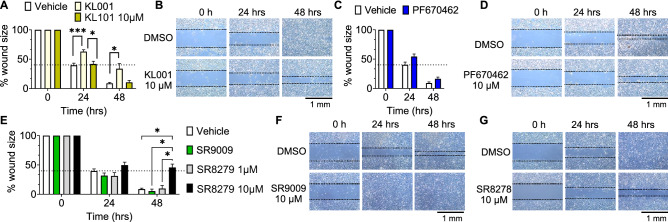
HTR-8 cell migration is regulated by ligands targeting CRY1/2 and NR1D1/2. Wound healing assays in HTR-8 cells, show that (**A**, **B**) KL001, a drug stabilizing CRY1/2, but not KL101, a drug stabilizing CRY1, reduced HTR-8 cell migration. (**C**, **D**) PF670462, a drug preventing PER1/2/3 degradation, did not impact HTR-8 cell migration. (**E**–**G**) The NR1D1/2 agonist SR9009, had no significant effect on migration, whereas the NR1D1/2 antagonist/inverse agonist, SR8278, decreased HTR-8 cell migration. All drugs were tested at 1 and 10 µM. The data were pooled if the two doses were not significantly different. (**B**, **D**, **F**, **G**) Illustrative wound healing assay images. Dotted line on histograms represents approximate wound area in control at 24 h. Data is expressed as % change in average wound size from 0 h ± SEM. N = 3–6, in duplicate. One-way ANOVA repeated measures, *, p < 0.05; ***, p < 0.001.

Based on the above PE-associated pathways, co-alteration of *CRY1*, *NR1D2,* and *PER3* in PE are predicted to impact cell migration. To assess whether CRY1, PER3 and NR1D2 regulate cell migration, we used the human-derived migratory trophoblast cell line HTR-8 to test drugs targeting CRY1, CRY1/2, PER1/2/3 and NR1D1/2. No drugs specifically targeting PER3 or NR1D2 are currently commercially available, thus only drugs targeting PER1/2/3 and NR1D1/2 were used for the study. Stabilizing/upregulating CRY1/2, using KL001 slowed HTR-8 migration at 24 h (Fig. 2A,B), whereas stabilizing/upregulating CRY1 with KL101, did not impact HTR-8 migration (Fig. 2A); these results suggest that CRY2 or CRY1/2 together impact trophoblast cell migration. Upregulating PER1/2/3 by preventing their degradation using PF670462 did not impact HTR-8 migration (Fig. 2C,D). In contrast, the NR1D1/2 agonist, SR9009, did not significantly change migration (Fig. 2E,F), whereas the NR1D1/2 antagonist/inverse agonist using SR8278, slowed migration of HTR-8 cells in a dose-dependent manner (Fig. 2E,G).

### CLOCK, NR1D2, and PER3 are downregulated in the term PE placenta—a subgroup analysis after removing patients diagnosed as PTB from GSE75010-157

PE frequently leads to PTB, particularly in severe cases of PE. To exclude the potential confounding effect of gestation length from PE-specific effects, we reanalyzed the data presented in Table 1 after removing patients diagnosed with PTB. In this reanalyzed data set, the expression of *CRY1* becomes non-significant between PE and non-PE, while *CLOCK* transcript is significantly lower in PE without PTB (Table 4, p = 0.0168 and FDR = 0.0952). Interestingly, both *NR1D2* (p = 0.0120, FDR = 0.0952) and *PER3* (p ≤ 0.0137 and FDR = 0.0952) transcripts in placenta are consistently lower in term PE (Table 4). This identifies *NR1D2* and *PER3* transcripts to be downregulated in PE, independent of gestation length. All other circadian gene transcripts in placental tissue were not significantly associated with term PE (p > 0.05 and FDR > 0.10, Table 4).

**Table 4:** Descriptive statistics of 17 candidate circadian genes’ expression levels in placenta from PE without PTB (Term-PE) vs. non-PE without PTB (Term) in GSE75010-157 dataset.

Gene	Term		Term-PE		p^b^	FDR^c^
	n	Mean^a^ (SD)	n	Mean (SD)		
*ARNTL*	42	7.22 (0.33)	31	7.39 (0.41)	0.0619	0.1754
*ARNTL2*	42	8.03 (0.37)	31	7.96 (0.39)	0.3919	0.5626
***CLOCK***	**42**	**9.06 (0.33)**	31	**8.73 (0.69)**	**0.0168**	**0.0952**
*CRY1*	42	8.55 (0.34)	31	8.52 (0.51)	0.7561	0.8034
*CRY2*	42	8.44 (0.24)	31	8.34 (0.30)	0.0943	0.2004
*NPAS1*	42	6.57 (0.37)	31	6.77 (0.46)	0.0449	0.1527
*NPAS2*	42	9.07 (0.29)	31	8.93 (0.34)	0.0724	0.1758
*NPAS3*	42	6.58 (0.44)	31	6.49 (0.41)	0.3971	0.5626
*NR1D1*	42	7.33 (0.34)	31	7.36 (0.33)	0.6501	0.7368
***NR1D2***	**42**	**8.92 (0.40)**	31	**8.61 (0.56)**	**0.0120**	**0.0952**
*PER1*	42	7.35 (0.26)	31	7.31 (0.38)	0.6164	0.7368
*PER2*	42	8.05 (0.32)	31	7.99 (0.29)	0.3812	0.5626
***PER3***	**42**	**7.34 (0.30)**	31	**7.16 (0.30)**	**0.0137**	**0.0952**
*RORA*	42	8.77 (0.32)	31	8.58 (0.41)	0.0308	0.1309
*RORB*	42	6.55 (0.58)	31	6.84 (0.97)	0.1535	0.2899
*RORC*	42	6.61 (0.35)	31	6.59 (0.36)	0.8158	0.8158
*TIMELESS*	42	7.78 (0.35)	31	7.72 (0.40)	0.5378	0.7033

^a^Mean of gene expression values, normalized and log2-transformed.

Bold values denote statistical significance (p < 0.05 and FDR < 0.10).

To identify the PE-associated pathways that correlate with the co-alteration of the *CLOCK, NR1D2,* and *PER3* genes as a whole in placenta, we first generated a linear combination of these three clock genes as a risk score, which was calculated as (10-*CLOCK*) + (11-*NR1D2*) + (9-*PER3*) based on the maximum values of the 3 genes, followed by the GSVA. The results demonstrate that a total of 41 term PE (PE without PTB)-associated pathways are significantly correlated with the *CLOCK, NR1D2,* and *PER3*-based risk score, among which 14 and 27 pathways are down- and up-regulated in the case group, respectively (the range of the absolute correlation coefficients is 0.25–0.53, all p ≤ 0.0344 and FDR ≤ 0.0520, Table S4).

Table 5 presents the top 10 down- and up-regulated pathways that are significantly correlated with *CLOCK, NR1D2,* and *PER3*-based risk score in placenta for term PE in GSE75010-157. Similarly, to better interpret the most represented pathways that are up- and down-regulated in PE in the sensitivity analysis, we manually collapsed the top-most regulated pathways into three broad categories: hypoxia-related pathways, membrane trafficking pathways, and autophagy-related pathways (Table 5). The increased hypoxia-related pathways include Cori Cycle, glycolysis and gluconeogenesis, computational model of aerobic glycolysis, HIF1 TF pathway, and reversible hydration of carbon dioxide. The decreased membrane trafficking pathways include intra Golgi traffic, RAB (Ras-associated binding) GEFs (GDP-GTP exchange factors), SNARE (soluble *N*-ethylmaleimide-sensitive factor attachment protein receptor) interactions in vesicular transport, RAB Regulation of Trafficking, and VxPx (a cilium localization motif) cargo targeting to cilium. The decreased autophagy-related pathways include pexophagy, nanoparticle triggered autophagic cell death, and autophagy in PE patients (Table 5).

**Table 5:** Top 10 pathways correlated with *CRY1, NR1D2* and *PER3*-based risk score in placenta (PE without PTB vs. non-PE without PTB) in GSE75010-157 dataset. Cut-off: p < 0.05 and FDR < 0.10.

Risk score*-correlated pathway	r	p	FDR
**Top 10 decreased pathways in PE without PTB**			
REACTOME_INTRA_GOLGI_TRAFFIC	− 0.53	< 0.0001	0.0007
REACTOME_RAB_GEFS_EXCHANGE_GTP_FOR_GDP_ON_RABS	− 0.47	< 0.0001	0.0007
KEGG_SNARE_INTERACTIONS_IN_VESICULAR_TRANSPORT	− 0.44	< 0.0001	0.0007
BIOCARTA_IGF1MTOR_PATHWAY	− 0.40	0.0004	0.0019
REACTOME_PEXOPHAGY	− 0.39	0.0005	0.0022
WP_NANOPARTICLE_TRIGGERED_AUTOPHAGIC_CELL_DEATH	− 0.39	0.0006	0.0023
REACTOME_RAB_REGULATION_OF_TRAFFICKING	− 0.35	0.0023	0.0068
REACTOME_VXPX_CARGO_TARGETING_TO_CILIUM	− 0.34	0.003	0.0078
REACTOME_TRANSLATION_OF_REPLICASE_AND_ASSEMBLY_OF_THE_REPLICATION_TRANSCRIPTION_COMPLEX	− 0.34	0.003	0.0078
WP_AUTOPHAGY	− 0.32	0.0055	0.0126
**Top 10 increased pathways in PE without PTB**			
WP_CORI_CYCLE	0.52	< 0.0001	0.0007
WP_GLYCOLYSIS_AND_GLUCONEOGENESIS	0.50	< 0.0001	0.0007
BIOCARTA_ACH_PATHWAY	0.46	< 0.0001	0.0007
WP_COMPUTATIONAL_MODEL_OF_AEROBIC_GLYCOLYSIS	0.45	< 0.0001	0.0007
PID_HIF1_TFPATHWAY	0.44	< 0.0001	0.0007
KEGG_ARACHIDONIC_ACID_METABOLISM	0.44	< 0.0001	0.0007
WP_ASPIRIN_AND_MIRNAS	0.42	0.0002	0.0012
WP_PHOTODYNAMIC_THERAPYINDUCED_HIF1_SURVIVAL_SIGNALING	0.40	0.0003	0.0016
REACTOME_ACTIVATION_OF_AMPK_DOWNSTREAM_OF_NMDARS	0.40	0.0003	0.0016
REACTOME_REVERSIBLE_HYDRATION_OF_CARBON_DIOXIDE	0.39	0.0006	0.0023

*Risk score = (10-*CLOCK*) + (11-*NR1D2*) + (9-*PER3*).

### Reduced CLOCK protein in term PE placenta

**Figure 3:**
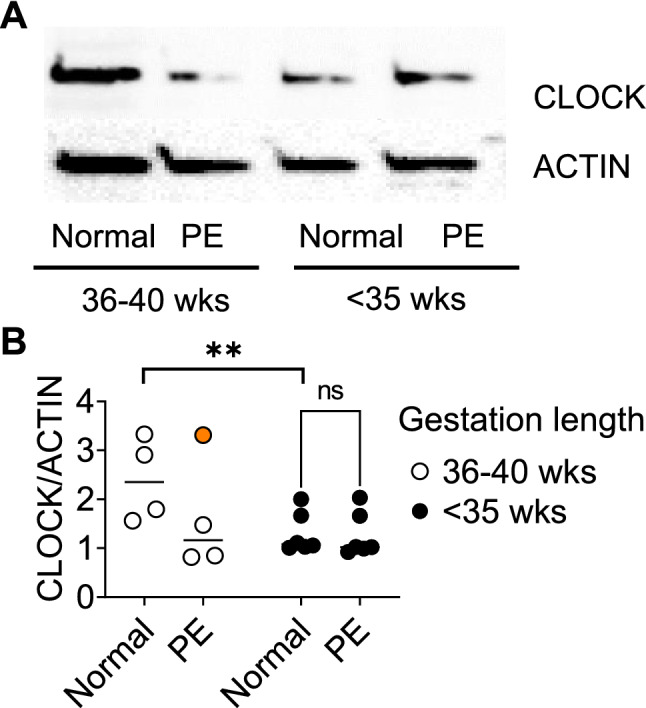
CLOCK protein is reduced in term PE placenta. (**A**) Example westernblot image for CLOCK and beta-ACTIN (ACTIN), of indicated human placenta samples. (**B**) Histogram of CLOCK/ACTIN in human placenta. Each dot represents a sample. Two-way ANOVA, **, p < 0.001. n = 3–6/group. Sample labeled in orange was an outlier and excluded from statistical analysis.

To determine if the small decrease in placenta *CLOCK* transcript in term PE samples (Table 4) translated into a change in CLOCK protein, we analyzed an independent cohort of 10 human placenta samples by westernblot (Fig. 3A). To obtain homogeneous group sizes, our “term” group was composed of late preterm + term placenta samples (35–40 weeks of gestation), whereas the PTB group was composed of placenta samples of < 35 weeks of gestation. Despite the small sample size (n = 3–6/group, total of 10 samples), we found that when excluding one term PE outlier (labeled in orange, Fig. 3B, sample not included in statistical analysis), term PE was associated with a significant reduction in CLOCK in the human placenta, as compared to term (Fig. 3B). Interestingly, the Two-way ANOVA showed that gestation length trended towards a reduction in CLOCK [F(1,5) = 6.020, p = 0.0577], supporting our previous work that identified that PTB correlated with reduced maternal blood *CLOCK* mRNA^56^.

## Discussion

In the present study, we found that the circadian gene transcripts for *CRY1, NR1D2,* and *PER3* in human placenta were robustly different between PE and non-PE across two independent datasets. Importantly, *NR1D2* and *PER3* were downregulated in placentas of PE patients independent of gestation length, *CLOCK* was downregulated in PE patients who delivered at term, and *CRY1* was upregulated in overall PE. Further analysis demonstrated that co-alteration of *CRY1, NR1D2,* and *PER3* transcripts were strongly associated with the risk of PE (the OR of PE was increased more than fivefold with the risk score > median). In vitro migration assays further identified a role of CRY1/2 and NR1D1/2 in trophoblast migration. Together, this identifies *CRY1, NR1D2,* and *PER3* as genes of particular interest in overall PE, as well as *CLOCK, NR1D2,* and *PER3* in term PE.

### CRY1, NR1D2, PER3 and CLOCK dysregulation in PE placenta

Clock genes are important in homeostatic regulation of cells and provide a molecular mechanism allowing tissues to anticipate daily changes in physiological functions. Despite the well-established role of circadian rhythms and clock genes in female reproductive function and pregnancy maintenance^37,39,42,43,69^, our understanding of the contribution of clock genes in placental development and function in relation to PE is in its infancy. Here, we find that among the 17 clock genes analyzed, only *CRY1*, *NR1D2* and *PER3* are consistently up- (*CRY1*) and down- (*NR1D2* and *PER3*) regulated in the PE placenta, independent of PE subtypes. It should be noted that the differential expression of 7 out of 17 studied clock genes (*ARNTL2*, *CLOCK, NPAS1*, *PER2*, *RORA, RORB*, and *TIMELESS*) were inconsistent between the two independent datasets. Specifically, *CLOCK* was significantly lower in PE patients than in controls from the dataset GSE75010-173, but not in the dataset GSE75010-157. Interestingly, our subgroup analysis demonstrated that after removing patients with PTB from the GSE750-157, the decreased expression of the *CLOCK* transcript in placenta of the term PE group became statistically significant, which is similar to the result from the dataset GSE75010-173. In contrast, removing patients with PTB from GSE75010-157 drives the *CRY1* level to be non-significant between term PE and term non-PE. These results suggest that alteration of the *CLOCK* transcript might be more specific to term PE, *CRY1* might be more specific to heterogeneous PE, whereas the expression patterns of *NR1D2* and *PER3* are more robustly associated with PE, independent of gestation length and PE subtype.

#### CRY1, NR1D2, and PER3 associated pathways and their potential role in the pathophysiology and etiology of PE through regulation of trophoblast migration

To further understand how dysregulation of *CRY1, NR1D2,* and *PER3* might impact placental function in relation to PE, we performed pathway analyses. The results revealed that among the top pathways that correlated with the co-alteration of the *CRY1/NR1D2/PER3* in overall PE (including preterm PE), the most enriched pathways include the hypoxia-related pathways, which were increased, and the cell migration and invasion pathways, which were decreased. Because abnormal trophoblast migration is a hallmark feature of PE^8,63–68^, we asked if pharmacological targeting of these clock proteins in the human migratory trophoblast cell line, HTR-8, would impact their migration. Due to the limited availability of drugs specifically targeting the studied proteins, we were unable to directly test the role of NR1D2 and PER3. Pharmacologically mimicking CRY1 upregulation, using KL101, did not significantly impact HTR-8 migration, whereas pharmacologically upregulating CRY1/2, using KL001, slowed HTR-8 migration. Although KL101 did not slow HTR-8 migration, studies in cancer cell lines have shown that down-regulation of CRY1 increases cell migration, indicating CRY1, at least under specific conditions, does regulate cell migration^70^. Future studies using primary migratory extravillous trophoblasts will determine if KL101 might slow migration in primary trophoblast cells, which more precisely reflect extravillous trophoblast migratory properties.

Previous work in mice and cell lines has shown a role of PER3 in cell migration, where *Per3* knock-down reduced migration^71–73^. Such reduction in cell migration when PER3 is low agrees with the predictions of our bioinformatics analysis. As no PER3 antagonist or drug downregulating PER3 are available, we asked if upregulation of PER1/2/3, using PF670462, would increase HTR-8 cell migration. In our assay, PF670462 did not impact HTR-8 migration. The lack of effect of PF670462 does not refute that low PER3 in HTR-8 cells would slow migration. Indeed, the fact that PF670462 upregulates PER1/2/3 might mask a specific effect of PER3 on HTR-8 cell migration; however, due to experimental constraints, we were unable to test whether PER3 downregulation would decrease HTR-8 migration. Another possibility is that PER3 might signal gestation length, as supported by our maternal blood study^56^, and preliminary GWAS study^74^, both of which suggest that deregulated or low *PER3* levels might be linked to gestation length and predispose women to PTB. Mechanisms that PER3 may engage to signal gestation length remain unknown.

The last target protein for our migration studies was NR1D2, and again our study was limited by the availability of drugs, as both drugs used targeted NR1D1/2. In agreement with our bioinformatics prediction, an NR1D1/2 agonist did not significantly impact migration, whereas NR1D1/2 antagonism/inverse agonism slowed migration of HTR-8 cells, indicating that the level of NR1D1/2 defines increased vs decreased trophoblast migration. Together, our migration assays, combined with published work indicate that reduced PER3, NR1D(1)/2, and potentially increased CRY1, might impair trophoblast migration, a well-established feature of PE. Future work establishing how these clock proteins regulate trophoblast migration and function will be key to understand if their deregulation is the cause or a symptom of PE.

While more rigorous studies are needed to further clarify the relationships among placental clock genes, PE, and these biological pathways, based on our current understanding of the pathogenesis of PE, the clock genes identified in this study are plausible candidates for the pathogenesis and etiology of PE. For example, we identified *NR1D2* and *PER3* to be downregulated in the PE placenta independent of gestation length, and we show functional migration data, supporting the idea that reduced PER3 and NR1D1/2 reduces trophoblast migration, a feature known to cause PE.

### CLOCK, NR1D2, and PER3 associated pathways and their potential role in the pathophysiology and etiology of term PE

In term PE patients, the most enriched pathways that were correlated with *CLOCK/NR1D2/PER3*-based risk score were hypoxia-related pathways and the membrane trafficking and autophagy-related pathways, which increased or decreased, respectively. Placental oxidative stress has been suggested to be central to the pathogenesis of preeclampsia^7,61,62^, whereas impaired trophoblast migration/invasion and spiral artery remodeling are also documented as hallmarks of PE^8,63–68^. While autophagy is regulated by membrane trafficking pathways in many species from yeast to mammals (reviewed by ^75,76^), the failure of placental autophagy has been reviewed as a risk factor of PE^77^. In contrast to known functional roles of NR1D2 and PER3 proteins outside their capacity to generate circadian rhythms, less is known about CRY1. The *CRY1* gene encodes a flavin adenine dinucleotide-binding protein that is best known for its role in altered sleep patterns. A dominant coding variation in the *CRY1* gene has been linked to familial delayed sleep phase disorder^78^, while PE has been associated with sleep-disordered breathing^79^. However, whether a similar mutation of the *CRY1* gene can be found in PE patients with sleep-disordered breathing and subsequently cause the change of *CRY1* transcript in placenta remains unclear.

More rigorous studies are needed to further clarify the relationships among placental clock genes, PE, and these biological pathways. Based on our current understanding of the pathogenesis of PE, the clock genes identified in this study are plausible candidates for the pathogenesis and etiology of PE. For example, we identified *NR1D2* and *PER3* to be downregulated in the PE placenta independent of gestation length. The nuclear receptor NR1D2 promotes expression of the inflammatory mediator, interleukin 6, a pathway that has been described to be involved in trophoblast hypoxia, arterial hypertension, placental inflammation, and autophagy in gestational diabetes mellitus^80–82^.

In support of our finding that term PE was associated with reduced *CLOCK* mRNA and protein expression in the term placenta, another study showed reduced CLOCK protein in human PE placenta^83^. Interestingly, this study also identified an increase in *CLOCK* mRNA, suggesting that mRNA processing or stability of *CLOCK* might be dysregulated in some cases of PE^83^. Like our study, these authors also used HTR-8 cells, which possess a functional molecular clock^35^ and respond to hypoxia by reducing *CLOCK* expression^83^.

### Strengths and limitations of the present study

Our study presents several strengths and limitations. We used 17 core clock gene candidates and conducted integrated pathway analysis in two independent datasets with > 75 individual samples per group in each. These analyses improved the biological plausibility of the gene-disease relationship as well as our understanding and interpretation of the final model. Our findings are robust thanks to the use of two independent datasets that have different definitions of PE and different race/ethnicity as well as the application of both ‘in silico and molecular approaches. The datasets analyzed are publicly available (NCBI GEO GSE75010-157 and GSE75010-173). On the other hand, these datasets do not report information of time-of-onset of PE, time-of-day, or time-of-year of sample collection. Future studies controlling for time-of-day and time-of-year in both clinical/epidemiological and laboratory settings will be important to further validate the presented findings. In addition, we also lack the information on the regions of the placentas from which the tissues were sampled for RNA extraction, as central vs. peripheral cotyledons, and basal plate vs. interior may be subject to different stressors and/or contain different cellular composition. More rigorous studies with the single-cell RNA sequencing technique are needed for further clarification.

## Conclusion

We here identify co-alterations of placental *CRY1/NR1D2/PER3* in overall PE (a mixture of PE subtypes including term and preterm PE) and *CLOCK/NR1D2/PER3* in term PE (excluding the samples from the patients with PTB) in human using bioinformatics analysis. We identify PE-associated pathways correlated with these clock genes, including increased hypoxia-related pathways as well as the decreased cell migration/invasion, autophagy, and membrane trafficking pathways in the PE placenta. We demonstrate that pharmacological targeting of CRY1/2 and NR1D1/2 in the human HTR-8 trophoblast cell line impacts migration and support the reduction of *CLOCK* protein in term PE through westernblot. Together these studies highlight *CRY1, CLOCK, NR1D2,* and *PER3* as novel genes of interest to study in placental development and function in relation to PE.

## Supplementary Information

Supplementary Tables.

Supplementary Table S3.

Supplementary Table S4.

Supplementary Information.

## Data Availability

The datasets analyzed during the current study are available from the corresponding author on reasonable request. The public data (GSE75010) underlying this article are available from the National Center for Biotechnology Information (NCBI) Gene Expression Omnibus (GEO) https://www.ncbi.nlm.nih.gov/geo/query/acc.cgi?acc=GSE75010.
