# Supplementary material for: Co-alterations of circadian clock gene transcripts in human placenta in preeclampsia
Source: Sci Rep. 2022 Oct 25;12:17856. doi: 10.1038/s41598-022-22507-3 (PMC9596722; doi:10.1038/s41598-022-22507-3)
Supplement: Supplementary file 1 — Supplementary Tables. [file 41598_2022_22507_MOESM1_ESM.docx]

**Table S1.** Maternal demographics of the participants in both overall non-PE and overall PE groups (GSE75010-157 dataset).

|  | **N (%)** | **Non-PE, n (%)** | **PE, n (%)** | **p*** |
| --- | --- | --- | --- | --- |
| Maternal race/ethnicity^1^: |  |  |  |  |
| Caucasian | 68 (44.4) | 33 (44.0) | 35 (44.9) | 0.9136 |
| Non-Caucasian | 85 (55.6) | 42 (56.0) | 43 (55.1) |  |
| Maternal age: |  |  |  |  |
| <30 years old | 37 (23.6) | 17 (22.1) | 20 (25.0) | 0.6663 |
| ≥30 years old | 120 (76.4) | 60 (77.9) | 60 (75.0) |  |
| Obesity before pregnancy^2^: |  |  |  |  |
| No | 115 (82.7) | 59 (84.3) | 56 (81.2) | 0.6258 |
| Yes (30<BMI<40) | 24 (17.3) | 11 (15.7) | 13 (18.2) |  |
| Previous miscarriage: |  |  |  |  |
| No | 117 (74.5) | 54 (70.1) | 63 (78.8) | 0.2153 |
| Yes | 40 (25.5) | 23 (29.9) | 17 (21.3) |  |

*Chi-square test. ^1^missing=4. ^2^missing=18.

**Table S2.** Study populations and sample sizes of 7 independent PE vs. Non-PE studies for microarray gene expression profiles (GSE75010-173 dataset).

| **GEO dataset ID** | **Population** | **Non-PE, n (%)** | **#PE, n (%)** | ***Total, n (%)** |
| --- | --- | --- | --- | --- |
| GSE4707 | Japan | 4 (4.2) | 10 (13.0) | 14 (8.1) |
| GSE10588 | Norway | 26 (27.1) | 17 (22.1) | 43 (24.9) |
| GSE24129 | Japan | 8 (8.3) | 8 (10.4) | 16 (9.2) |
| GSE25906 | US | 37 (38.5) | 23 (29.9) | 60 (34.7) |
| GSE30186 | China | 6 (6.3) | 6 (7.8) | 12 (6.9) |
| GSE43942 | China | 7 (7.3) | 5 (6.5) | 12 (6.9) |
| GSE44711 | Canada | 8 (8.3) | 8 (10.4) | 16 (9.2) |
| Total |  | 96 (100.0) | 77 (100.0) | 173 (100.0) |

*Proportion of all patients (PE+Non-PE) by country: Japan=17.3% (30/173), China=13.9% (24/173), Norway=24.9% (43/173), US=34.7% (60/173), Canada=9.2% (16/173).

#Proportion of PE patients by country among all PE cases: Japan=18/77=23.4%, China=11/77=14.3%, Norway=17/77=22.1%, US=23/77=29.9, Canada=8/77=10.4%.

**Table S3.** All significant common pathways correlated with 3 common clock genes-based risk score in placenta (overall PE vs. overall non-PE).

**Table S4.** All significant pathways correlated with 3 clock genes-based risk score in placenta (PE without PTB vs. non-PE without PTB).
