# Supplementary material for: Co-alterations of circadian clock gene transcripts in human placenta in preeclampsia
Source: Sci Rep. 2022 Oct 25;12:17856. doi: 10.1038/s41598-022-22507-3 (PMC9596722; doi:10.1038/s41598-022-22507-3)
Supplement: Supplementary file 4 — Supplementary Information. [file 41598_2022_22507_MOESM4_ESM.docx]

**Co-Alterations of Circadian Clock Gene Transcripts in Human Placenta in Preeclampsia**

Guoli Zhou, Emily Winn, Duong Nguyen, Eric P. Kasten, Margaret G. Petroff, Hanne M. Hoffmann


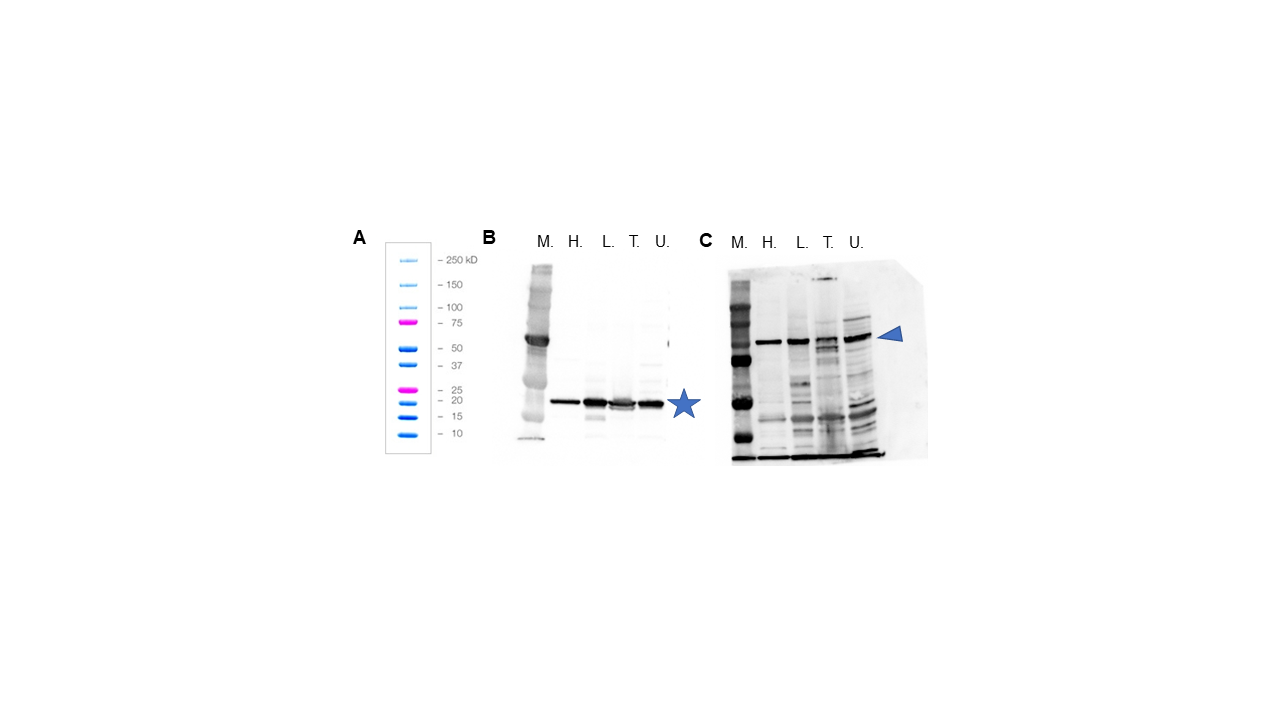


**Figure S1.** Validation of CLOCK antibody for human samples.

Representative western blot showing A) molecular weight marker and westernblot image for B) beta ACTIN (ACTIN, ~42 kDa, band indicated with a star) and C) CLOCK protein (~95 kDa, band indicated with an arrow). The used CLOCK antibody has previously been validated in mouse^1–3^. To confirm this antibody also recognizes CLOCK in human samples, we compared CLOCK staining in mouse and human samples. Abbreviations: M: Molecular weight marker, H: Human placenta, L: Mouse lung, T: mouse thymus, and U: mouse uterus (U). These westernblots were used to validate initial specificity of the antibodies, and the images were not quantified. Although these images are overexposed, these images were only used for initial antibody validation, and were not quantified, rendering the over-exposure less of a concern. As can clearly be seen in C, the CLOCK band is at the proper molecular weight in human and mouse samples, validating the CLOCK antibody for the use in human samples.

**
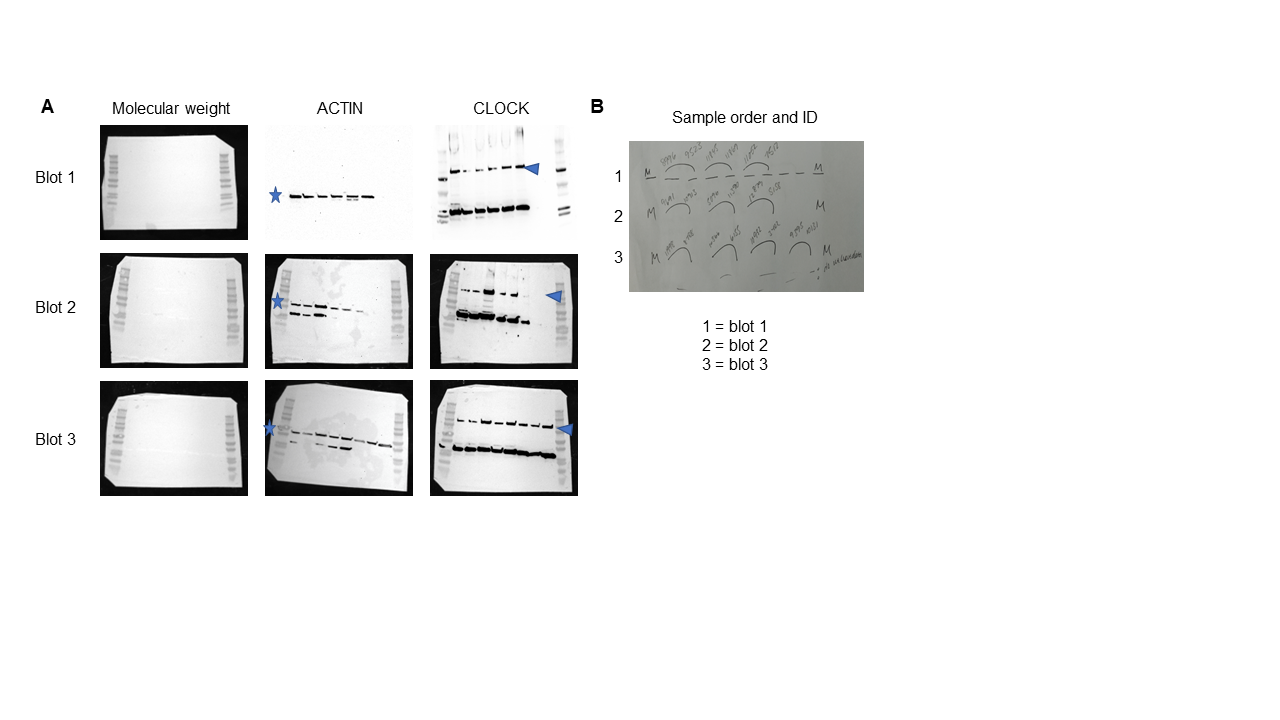
Figure S2. Original westernblot images**.

A) Representative full length westernblot images for Figure 3 for indicated antibodies. The molecular weight marker is shown to the left for each blot. Middle panel shows beta ACTIN (ACTIN) at ~42 kDa (band indicated with a start) and right panel shows CLOCK protein at ~95 kDa (band indicated with a triangle). To allow better visualization of ACTIN and CLOCK on Blot 1, images were inverted and enhanced for contrast in Image J. B) Gel organization for Blots 1, 2 and 3, M indicates position of molecular wight marker.

**References**

1. Qi, F. *et al.* The Clock-Controlled lncRNA-AK028245 Participates in the Immune Response via Immune Response Factors OTUD7B and A20. *J. Biol. Rhythms* **35**, 542–554 (2020).

2. Dan, H., Ruan, T. & Sampogna, R. V. Circadian Clock Regulation of Developmental Time in the Kidney. *Cell Rep.* **31**, 107661 (2020).

3. Yao, Y. *et al.* Non-invasive 40-Hz Light Flicker Ameliorates Alzheimer’s-Associated Rhythm Disorder via Regulating Central Circadian Clock in Mice. *Front. Physiol.* **11**, (2020).
